#  Novel Oxadiazole Thioglycosides as Potential Anti-*Acinetobacter *Agents

**Published:** 2016

**Authors:** Karim Akbari Dilmaghani, Fazel Nasuhi Pur, Majid Mahammad pour, Jafar Mahammad nejad

**Affiliations:** a *Department of Chemistry, Faculty of Science, Urmia University, Urmia, Iran. *; b *Health Technology Incubator Center, Urmia University of Medical Sciences, Urmia, Iran.*

**Keywords:** 1, 3, 4-Oxadiazole-2-thione, β-Pyranosyl, Thioglycoside, Nucleoside, *in-vitro*, *Acinetobacter calcoaceticus*, Antiproliferative activity

## Abstract

The glycosylation of 1,3,4-oxadiazole-2-thiones has been performed with peracetylated β-pyranosyl bromide in the presence of potassium carbonate. Deprotection of acetylated thioglycosides was necessary for increasing their antibacterial effects. The structures of nucleosides were confirmed by ^1^H NMR, ^13^C NMR and HRMS. The anomeric protons of nucleosides c_1–4_ were assigned to the doublet, confirming the β-configuration. The synthesized compounds were tested for their antimicrobial activity against *Acinetobacter calcoaceticus* (Gram-negetive) strain *in-vitro* in comparison with Ampicillin as a reference drug which is normally used for treating such infections. The synthetic compounds showed different inhibition zones against tested bacterial strain. Thioglycoside derivatives of 1,3,4-oxadiazole-2-thiones (c set) were more active against *Acinetobacter calcoaceticus *ATCC 23055 than “parent” 1,3,4-oxadiazole-2-thiones (a set), confirming the relation between glyco-conjugation and increasing of antiproliferative activity of antibiotic agents. The best result belonged to nucleoside bearing 2-furyl moiety in its heterocyclic nucleus (c_4_). The existence of *m*-PhNO_2_ group as Ar in structures of a set and their corresponding sugar derivatives decreased the antibacterial activity of them in comparison with the rest of synthetic compounds.

## Introduction

The resistance of infective bacteria to present antibiotics demands research assigned to the discovery of new drugsin the antibacterial drug field. The majority of carbohydrates found in nature or biological systems exist as glycoconjugates in which the monosaccharide units are joined via O-, N-, or S-glycosidic bonds. Thioglycosides have received considerable attention, because they are widely employed as biological inhibitors, inducers and ligandsfor affinity chromatography of carbohydrate-processing enzymes and proteins (1-8). They have excellent chemoselectivity in glycosylation processes as both donors and acceptorsparticularly via reaction processesthat involve active and latent glycosylation protocols (9). The thioglycosyl heterocycles are sufficiently stable under a variety of reaction conditions and have the ability to be readilyconverted into a variety of other functionalities (10, 11). Multivalent display of carbohydrates is frequently used as a method to increase affinities in various contexts such as the binding of bacteria, bacterial toxins, galectins and other lectins (12-24). These properties may affect medicinal effect of antibiotic agents.

On the other hand, oxadiazole derivatives, which belong to an important group of heterocyclic compounds, have been thesubject of extensive study in the recent past. Numerous reports have highlighted their chemistry anduse. Diverse biological activities, such as antiinflamatory, antitumor, antimicrobial and anticonvulsant, have been found to be associated with oxadiazole derivatives (25-28). Moreover, sulfur-containing heterocycles represent an important group of sulfur compounds that are promising for use in practical applications.

**Figure 1:**
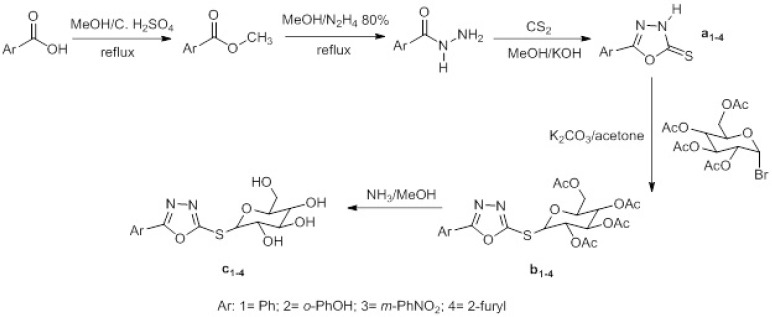
General synthetic pathway for the synthesis of thioglycosyl oxadiazoles

**Table 1:** *In-vitro* antibacterial activity of synthetic compounds against *Acinetobacter Calcoaceticus *ATCC 23055 (concentration = 10 µg/µL

**Compound**	**Diameter of inhib. zone (mm)**	**Compound**	**Diameter of inhib. zone (mm)**
**a**_1_	19	**c**_1_	28
**a**_2_	18	**c**_2_	29
**a**_3_	16	**c**_3_	25
a_4_	**22**	c_4_	**31**

Therefore, it is interesting to report the synthesis of a new series of compounds in which the glycosyl moieties have been used as carriers for the heterocycles having the oxadiazole ring.

In our previous work (29), we reported the synthesis antibacterial properties of new series of thioglycoside derivatives of 1,2,4-triazole-5-thiones, whereas in the present work, we report the synthesisof new groups of anti-*Acinetobacter calcoaceticus* agents in which 1,3,4-oxadiazole-2-thiones moiety is coupled to monosaccharide unit.

## Experimental

^1^H and ^13^C NMR spectra were recorded on a Bruker AVANCE-300 spectrometer at 300 and 75 MHz, respectively in CDCl_3 _using TMS as the internal standard. High-resolutionmass spectra were obtained with a HPLC-Q-TOF system equipped with Q-TOF micromass spectrometer (dual ESI). Melting points were measured on a Philip Harris C4954718apparatus without calibration. Optically active samples were analyzed by EHARTNACKapparatus (Paris, France) at 20 °C in dichloromethane. Thin layer chromatography (TLC) analyses were carried out on silica gel plates. All chemicals were purchased from Merckand used as received.

*5-phenyl-1,3,4-oxadiazole-2(3H)-thione (a*_1_ *)*

Yield: 74%; mp 158-160 °C; ^1^H NMR spectrum, (300 MHz, CDCl_3_), δ, ppm, (J, Hz): ): 7.52 (m, 3H, ArH), 7.96 (d, J = 6.9, 2H, ArH) 10.75 (bs, 1H, N-H); ^13^C NMR spectrum, (75 MHz, CDCl_3_), δ, ppm: 122.90, 126.47, 129.85, 132.67, 160.90 (Ar), 177.88 (C=S).

*5-(2-hydroxyphenyl)-1,3,4-oxadiazole-2(3H)-thione (a*_2_ *)*

Yield: 84%; mp 162-164 °C; ^1^H NMR spectrum, (300 MHz, CDCl_3_), δ, ppm, (J, Hz): ): 7.10 (m, 2H, ArH), 7.49 (t, 1H, J = 7.1, ArH), 7.72 (2, 1H, J = 8.1, ArH), 8.39 (bs, 1H, OH), 11.34 (bs, 1H, N-H); ^13^C NMR spectrum, (75 MHz, CDCl_3_), δ, ppm: 109.79, 111.17, 119.84, 129.50, 133.87, 156.78, 160.27 (Ar), 177.47 (C=S).

*5-(3-nitrophenyl)-1,3,4-oxadiazole-2(3H)-thione (a*_3_ *)*

Yield: 78%; mp 167-168 °C; ^1^H NMR spectrum, (300 MHz, CDCl_3_), δ, ppm, (J, Hz): ): 7.40-7.60 (m, 3H, ArH), 7.94 (d, J = 7.8, 1H, ArH), 11.10 (bs, 1H, OH); ^13^C NMR spectrum, (75 MHz, CDCl_3_), δ, ppm: 120.93, 124.43, 126.85, 131.76, 132.44, 148.53, 159.15 (Ar), 178.04 (C=S)

*5-(2-furyl)-1,3,4-oxadiazole-2(3H)-thione (a*_4_ *)*

Yield: 69%; mp 152-155 °C; ^1^H NMR spectrum, (300 MHz, CDCl_3_), δ, ppm, (J, Hz): ): 6.62 (bs, 1H, Furyl), 7.18 (bs, 1H, Furyl), 7.66 (bs, 1H, Furyl), 11.38 (bs, 1H, N-H); ^13^C NMR spectrum, (75 MHz, CDCl_3_), δ, ppm: 113.05, 115.37, 137.99, 147.61, 153.90 (Ar), 177.10 (C=S).

*2-phenyl-5-(2,3,4,6-tetra-O-acetyl-β-D-1-thio-glucopyranose)-1,3,4-oxadiazole (b1)*

Yield: 64%; mp 102-105 °C; ^1^H NMR spectrum, (300 MHz, CDCl_3_), δ, ppm, (J, Hz): ): 1.95 (s, 3H, OAc), 2.04 (s, 3H, OAc), 2.09 (s, 3H, OAc), 2.18 (s, 3H, OAc), 3.88-3.99 (m, 1H, H-6a), 4.12-4.20 (m, 1H, H-6b), 4.27-4.30 (m, 1H, H-5), 5.14-5.71 (m, 3H, H-2, -3, -4), 5.96 (d, 1H, J_1,2 _= 9.3, H-1), 7.52 (m, 3H, ArH), 7.94-8.04 (m, 2H, ArH); ^13^C NMR spectrum, (75 MHz, CDCl_3_), δ, ppm: 20.56 (2C), 20.72 (2C) (4 × OCOCH_3_), 61.55 (C-6), 67.56 (C-4), 69.76 (C-2), 73.52 (C-3), 74.71 (C-5), 83.42 (C-1), 121.93, 126.80, 129.18, 132.03, 132.79 (Ar), 166.52 (C−S), 168.93, 168.37, 170.06, 170.58 (4 × OCOCH_3_).

*2-(2-hydroxyphenyl)-5-(2,3,4,6-tetra-O-acetyl-β-D-1-thio-glucopyranose)-1,3,4-oxadiazole (b2)*

Yield: 66%; mp 99-101 °C; ^1^H NMR spectrum, (300 MHz, CDCl_3_), δ, ppm, (J, Hz): ): 2.03 (s, 3H, OAc), 2.04 (s, 3H, OAc), 2.06 (s, 3H, OAc), 2.10 (s, 3H, OAc), 3.88-3.92 (m, 1H, H-6a), 4.13-4.17 (m, 1H, H-6b), 4.27-4.32 (m, 1H, H-5), 5.14-5.37 (m, 3H, H-2, -3, -4), 5.50 (d, 1H, J_1,2 _= 9.6, H-1), 7.02 (t, 1H, J = 7.8, ArH), 7.13 (d, 1H, J = 8.4, ArH), 7.47 (t, 1H, J = 6.9, ArH), 7.72 (d, 1H, J = 7.8, ArH), 9.87 (bs, 1H, OH); ^13^C NMR spectrum, (75 MHz, CDCl_3_), δ, ppm: 20.54 (2C), 20.60 (2C) (4 × OCOCH_3_), 61.56 (C-6), 67.72 (C-4), 69.74 (C-2), 73.46 (C-3), 76.62 (C-5), 83.33 (C-1), 107.58, 117.66, 120.11, 126.54, 134.04, 157.34, 160.02 (Ar), 165.92 (C−S), 169.34, 169.43, 169.966, 170.54 (4 × OCOCH_3_).

*2-(3-nitrophenyl)-5-(2,3,4,6-tetra-O-acetyl-β-D-1-thio-glucopyranose)-1,3,4-oxadiazole (b3)*

Yield: 58%; mp 112-113 °C; ^1^H NMR spectrum, (300 MHz, CDCl_3_), δ, ppm, (J, Hz): ): 2.04 (s, 3H, OAc), 2.06 (s, 3H, OAc), 2.09 (s, 3H, OAc), 2.10 (s, 3H, OAc), 3.88-4.05 (m, 1H, H-6a), 4.14-4.21 (m, 1H, H-6b), 4.28-4.33 (m, 1H, H-5), 5.16-5.67 (m, 3H, H-2, -3, -4), 5.97 (d, 1H, J_1,2 _= 9.3, H-1), 7.75 (t, J = 7.8, 1H, ArH), 8.27-8.43 (m, 2H, ArH), 8.84 (d, J = 8.1, 1H, ArH); ^13^C NMR spectrum, (75 MHz, CDCl_3_), δ, ppm: 20.53 (2C), 20.65 (2C) (4 × OCOCH_3_), 61.50 (C-6), 67.70 (C-4), 69.71 (C-2), 73.49 (C-3), 74.83 (C-5), 83.32 (C-1), 121.80, 124.93, 126.36, 127.03, 130.52, 132.21, 148.67 (Ar), 164.50 (C-S), 169.33, 169.44, 169.97, 170.53 (4 × OCOCH_3_).

*2-(2-furyl)-5-(2,3,4,6-tetra-O-acetyl-β-D-1-thio-glucopyranose)-1,3,4-oxadiazole (b4)*

Yield: 72%; mp 107-109 °C; ^1^H NMR spectrum, (300 MHz, CDCl_3_), δ, ppm, (J, Hz): ): 1.97 (s, 3H, OAc), 2.04 (s, 3H, OAc), 2.07 (s, 3H, OAc), 2.10 (s, 3H, OAc), 3.85-4.00 (m, 1H, H-6a), 4.13-4.21 (m, 1H, H-6b), 4.27-4.32 (m, 1H, H-5), 5.24 (t, 1H, J_1,2_=J_2,3 _= 9.3, H-2), 5.42 (t, 1H, J_2,3_=J_3,4 _= 9.3, H-4), 5.61 (t, 1H, J_2,3_=J_3,4_ = 9.3, H-3), 6.34 (d, 1H, J_1,2 _= 9.3, H-1), 6.61 (bs, 1H, Furyl), 7.17 (bs, 1H, Furyl), 7.66 (bs, 1H, Furyl); ^13^C NMR spectrum, (75 MHz, CDCl_3_), δ, ppm: 20.49 (2C), 20.70 (2C) (4 × OCOCH_3_), 61.54 (C-6), 67.51 (C-4), 69.40 (C-2), 73.04 (C-3), 74.75 (C-5), 83.17 (C-1), 112.36, 116.17, 137.41, 146.80, 152.37 (Ar), 159.98 (C-S), 168.97, 169.28, 170.04, 170.56 (4 × OCOCH_3_).

*2-phenyl-5-(β-D-1-thio-glucopyranose)-1,3,4-oxadiazole (c1)*

Yield: 33%; mp 108-110 °C; [α]_D_^20^= 3° (*c *= 1.0, CH_2_Cl_2_). ^1^H NMR spectrum, (300 MHz, CDCl_3_), δ, ppm, (J, Hz): ): 3.92-4.03 (m, 1H, H-6a), 4.12-4.22 (m, 1H, H-6b), 4.27-4.30 (m, 1H, H-5), 4.35-4.85 (m, 4H, OH), 5.12-5.70 (m, 3H, H-2, -3, -4), 5.99 (d, 1H, J_1,2 _= 9.3, H-1), 7.50 (m, 3H, ArH), 7.94-8.02 (m, 2H, ArH); ^13^C NMR spectrum, (75 MHz, CDCl_3_), δ, ppm: 61.76 (C-6), 67.64 (C-4), 69.26 (C-2), 73.59 (C-3), 74.78 (C-5), 83.37 (C-1), 122.23, 126.88, 129.40, 132.09, 132.99 (Ar), 166.45 (C−S); HRMS spectrum (ESI), *m/z*: Calculated, 340.0729. C_14_H_16_N_2_O_6_S [M+H]^+^. Found, 341.0817.

*2-(2-hydroxyphenyl)-5-(β-D-1-thio-glucopyranose)-1,3,4-oxadiazole (c2)*

Yield: 46%; mp 144-145 °C; [α]_D_^20^= 4° (*c *= 1.0, CH_2_Cl_2_). ^1^H NMR spectrum, (300 MHz, CDCl_3_), δ, ppm, (J, Hz): ): 3.86-3.97 (m, 1H, H-6a), 4.11-4.16 (m, 1H, H-6b), 4.26-4.32 (m, 1H, H-5), 4.37-4.83 (m, 4H, OH), 5.13-5.37 (m, 3H, H-2, -3, -4), 5.57 (d, 1H, J_1,2 _= 9.6, H-1), 7.03 (t, 1H, J = 7.8, ArH), 7.12 (d, 1H, J = 8.4, ArH), 7.49 (t, 1H, J = 6.9, ArH), 7.74 (d, 1H, J = 7.8, ArH), 9.97 (bs, 1H, OH); ^13^C NMR spectrum, (75 MHz, CDCl_3_), δ, ppm: 62.46 (C-6), 68.02 (C-4), 69.86 (C-2), 73.65 (C-3), 76.69 (C-5), 83.04 (C-1), 109.18, 117.36, 121.01, 126.94, 133.84, 157.86, 160.23 (Ar), 165.57 (C−S); HRMS spectrum (ESI), *m/z*: Calculated, 356.0678. C_14_H_16_N_2_O_7_S [M+H]^+^. Found, 357.0762.

*2-(3-nitrophenyl)-5-(β-D-1-thio-glucopyranose)-1,3,4-oxadiazole (c3)*

Yield: 39%; mp 122-124 °C; [α]_D_^20^= -2° (*c *= 1.0, CH_2_Cl_2_). ^1^H NMR spectrum, (300 MHz, CDCl_3_), δ, ppm, (J, Hz): ): 3.84-4.01 (m, 1H, H-6a), 4.17-4.25 (m, 1H, H-6b), 4.24-4.33 (m, 1H, H-5), 4.36-4.85 (m, 4H, OH), 5.16-5.68 (m, 3H, H-2, -3, -4), 6.07 (d, 1H, J_1,2 _= 9.3, H-1), 7.78 (t, J = 7.8, 1H, ArH), 8.27-8.46 (m, 2H, ArH), 8.81 (d, J = 8.4, 1H, ArH); ^13^C NMR spectrum, (75 MHz, CDCl_3_), δ, ppm: 62.20 (C-6), 67.74 (C-4), 69.79 (C-2), 73.67 (C-3), 74.35 (C-5), 83.44 (C-1), 121.89, 123.90, 126.86, 127.43, 131.82, 132.28, 149.63 (Ar), 165.22 (C-S); HRMS spectrum (ESI), *m/z*: Calculated, 385.0580. C_14_H_15_N_3_O_8_S [M+H]^+^. Found, 386.0665.

*2-(2-furyl)-5-(β-D-1-thio-glucopyranose)-1,3,4-oxadiazole (c4)*

Yield: 52%; mp 111-113 °C; [α]_D_^20^= -4° (*c *= 1.0, CH_2_Cl_2_). ^1^H NMR spectrum, (300 MHz, CDCl_3_), δ, ppm, (J, Hz): ): 3.82-4.08 (m, 1H, H-6a), 4.17-4.23 (m, 1H, H-6b), 4.24-4.39 (m, 1H, H-5), 4.35-4.89 (m, 4H, OH), 5.27 (t, 1H, J_1,2_=J_2,3 _= 9.3, H-2), 5.45 (t, 1H, J_2,3_=J_3,4 _= 9.3, H-4), 5.69 (t, 1H, J_2,3_=J_3,4_ = 9.3, H-3), 6.31 (d, 1H, J_1,2 _= 9.3, H-1), 6.66 (bs, 1H, Furyl), 7.17 (bs, 1H, Furyl), 7.58 (bs, 1H, Furyl); ^13^C NMR spectrum, (75 MHz, CDCl_3_), δ, ppm: 60.94 (C-6), 66.98 (C-4), 69.87 (C-2), 73.54 (C-3), 74.85 (C-5), 83.63 (C-1), 112.30, 117.20, 137.83, 146.88, 152.52 (Ar), 162.07 (C-S); HRMS spectrum (ESI), *m/z*: Calculated, 330.0522. C_12_H_14_N_2_O_7_S [M+H]^+^. Found, 331.0609.

*Bacterial Strain*

The antibacterial activity of compounds was assayed with our previous published 

method (30). The antibacterial activity of the compounds was tested against Gram-negative strain of *Acinetobacter calcoaceticus *ATCC 23055.

## Resultsand Discussion

Thioglycosilation was performed according our previous published method (29). 1-bromide sugar and 1,3,4-oxadiazole-2-thione nuclei a_1-4_ were synthesized to the literature procedures (29,31). Deprotection of acetylated nucleosides was performed to the literature procedure (32). The synthesis of the final nucleosides is depicted in Figure 1.

The structure of thioglycosides was confirmed by appropriate spectroscopic methods such as^1^HNMR,^13^C NMR, and high resolution mass spectroscopy (HRMS). The anomeric protons of nucleosides c_1–4_ were assigned to the doublet at 5.57–6.31 ppm with J_1,2_=9.3–9.6 Hz, confirming theβ-configuration.

In our recent published research (29), there was not any significant change in the antibacterial effect of the acetylated final thioglycosides in comparison with the parent heterocyclic nuclei. Therefore, in the present work, the deacetylated nucleosides c_1-4 _have been compared to the parent heterocyclic nuclei a_1-4 _against *Acinetobacter calcoaceticus*.

The in vitro antibacterial activity of the synthesized compounds in DMSO against *Acinetobacter calcoaceticus *is shown in Tables 1. Diameter of inhibition zone for ampicillin as reference drug is 18 mm for 10 µg/µL concentration of drug in the same test conditions.

As shown in the Table 1. these compounds showed higher antibacterial effects in comparison with Ampicillin (18 mm) which is normally used for treating such infections.

In general, compounds from **c** set (nucleosides) showed more antimicrobial activity than the other set. Thioglycoside derivatives of 1,3,4-oxadiazole-2-thiones (c set) were more active against *Acinetobacter calcoaceticus *ATCC 23055 than “parent” 1,3,4-oxadiazole-2-thiones (a set), confirming the relation between glyco-conjugation and increasing of antiproliferative activity of antibiotic agents. The best results in the tables belonged to **c**_4_that showing high activity against *A. calcoaceticus *(31 mm).

Going over the structure of these synthetic compounds confirmed that the existence of 2-furyl instead of *m*-PhNO_2_ group as Ar increased their antibacterial activity against *A. calcoaceticus*. The existence of *m*-PhNO_2_ group as Ar in structures of a set and their corresponding sugar derivatives decreased the antibacterial activity of them in comparison with the rest of synthetic compounds.
